# Medicine in Eighteenth-Century Bristol

**Published:** 1950-01

**Authors:** H. J. Orr-Ewing

**Affiliations:** Physician, United Bristol Hospitals


The Bristol
Medico-Chirurgical Journal

*A Journal of the Medical Sciences for the*

West of England and South Wales

"*Scire est nescire, nisi id me*
*Scire alius sciret.*"

JANUARY, 1950

### MEDICINE IN EIGHTEENTH-CENTURY BRISTOL

The Presidential Address, delivered on Wednesday, 12th October, 1949, at the opening of the Seventy-first Session of the Bristol Medico-Chirurgical Society

BY

DR. H. J. ORR-EWING, F.R.C.P.
*Physician, United Bristol Hospitals*

The President introduced his remarks by a short survey of the social and economic conditions in Bristol in the middle of the eighteenth century, with acknowledgments to Latimer's *Annals of Bristol in the eighteenth century*, Munro Smith's *History of the Bristol Royal Infirmary*, and other sources. He quoted from *English Social History*: " Good doctoring rather than better social conditions was the cause of the great increase in population which took place "; and continued:

I can't see myself that there *was* all this great improvement in doctoring. It seems to have been still largely under the influence of Mumbo-Jumbo.

What diseases did our predecessors treat? It is difficult to be precise, for their methods of diagnosis were necessarily so imperfect that they arrived very often at no " diagnosis " in our sense of the word. Scan the eighteenth-century records of patients examined at the B.R.I. and you will find that in the majority of cases the "diagnosis" recorded is merely the predominant symptom. Thus "cough" figures very largely in the winter months. "Suppression of menses", and "anaemia" are two other favourite entries. Phthisis is not infrequently given as the cause for the patient's attendance, and one wonders! Without any means of auscultation more delicate than the human ear applied to the chest, without a stethoscope, without X-rays, one wonders how they were sure. But doubtless the signs of the wasting disease accompanied by cough, "hectic" temperature and phthisical facies made the probability only too likely. Another feature which leaps to the eye in these books of attendances is the wonderful way in which they cured their patients, from cough, fever, suppression of menses, anaemia—90 per cent. of the entries have "cured" against them. A few of the cachectics and phthisics have "relieved", and some of them are recorded as "non-attendance": only too often a euphemism, one fears, for death. On the whole one can guess pretty well what diseases would be prevalent in a city over-crowded with very many indigent poor and no public health service. Typhoid was common, cholera often put in its dread appearance, plague was not unknown (but rare in the eighteenth century and then brought in from the East by ship). Small-pox, typhus and enteric raged through a population little addicted to cleanliness. Hospital gangrene stalked the wards and carried off many victims after even the smallest and least dangerous operations according to our view. The nursing was done by a body of women almost totally uneducated and illiterate, many of them drunken sluts, of whom, years later Sairey Gamp was only too apt a picture.

And with what remedies did our forefathers attack the prevalent disorders? Well, the apothecaries were the most ardent and venturesome drug dispensers the world perhaps has ever seen. Then, as now, the backbone of the profession was the apothecary who corresponded in many ways to the G.P. of to-day. These people appear to have done almost all the work of the profession. They saw all the ordinary patients, prescribed for them the most colossal amount of drugs, had them made up and sent round. They worked tremendously hard and their assistants worked even harder. The apothecaries started, originally, as people who in their grocers' shops sold drugs : but for more than 100 years at this time they had been separated from such companions, a considerable period (120 years) before similar divorce of the barbers from the surgeons. There was at this time no diploma for apothecaries. Apparently anyone who wished could set up in a shop where drugs were sold and medical stores (such as they were) dispensed. They started seeing patients at home. They could not charge for attendance but only for medicines supplied, a space frequently being left below the account where it was stated " attendance, what you please ". It is recorded of Broderip, one of the most successful of eighteenth-century apothecaries, that on one occasion he was given a douceur of £50 in addition to his account of £30 for drugs. Originally the apothecaries were kept very much under the control of the Royal College of Physicians, who limited their powers of dealing with patients. But in the eighteenth century they had largely succeeded in escaping from this position. Although they were greatly despised by the Physicians yet before the end of the century the latter had found it was much wiser to keep on good terms with them. The best apothecaries seem to have been those who were fortunate enough to be assistants in the Infirmary, where they served an apprenticeship which varied in time from one year to several years, during which they saw a great number of patients and dispensed many thousands of prescriptions. Then they set up for themselves. The amount of drugs they dispensed was almost incredible. R. Smith relates that a Mr. Archer who lived in his family paid £350 in three years for medicines. Even the endocrinologists nowadays would not cost more with their remedies if one reckons the value of money then and now. Broderip, one of the busiest apothecaries, was said for years to have made £4,000 a year. One year he booked £5,993 but £1,900 was bad debts (worse than at present !). His carriage was said to have stopped at sixty houses in a day. He inoculated for smallpox (three to five guineas), he bled people, his assistants dispensed drugs up to midnight. One family took on their summer holiday 200 tonic draughts and 1,000 pills.

Some of these people did not hesitate in the least to advertise.

In 1760 James Grace advertised in the Bristol papers that he had fitted up his shop opposite the pump in Peter Street. " County people afflicted with any sort of disease by sending the state of their several complaints will be fitted with such medicines as shall speedily cure them." One man advertised that " he certainly cures cancerous complaints, scurvy, rheumatism, dropsy and gravel ". (*How* I wish I could!)

In 1754 there were twenty-nine apothecaries in Bristol, in 1793 there were thirty-five. We are not informed how many physicians.* The apothecaries seem to have dressed more or less like ordinary folk without distinctive marks (different from the physicians). They lived scattered all over the city. Broderip the famous one, when he was making £4,000 per annum, built a house out towards Westbury over Durdham Down: but generally doctors " lived over the shop " and not a bad idea either. A great deal of the medicines they dispensed was what we should call the merest excipients and placebos: but one man used two cwt. of Epsom salts per annum. In three or four months one boy had sent in 469 draughts besides medicines. One patient was ordered twelve draughts a day. How far we have travelled!

Four shillings was the usual charge for a packet of twelve powders, chiefly rhubarb, chalk and a very small dose of antimony. During the eighteenth century the practice of drug taking reached the " all time high " and by 1810 the physicians and druggists had ousted the apothecaries and the practice then died down.

The physicians would have felt extremely irritated at being taken second in my list, for they considered themselves in every way immensely superior to the apothecaries. They were all of them persons with university degrees from Oxford or Cambridge: and a little later Edinburgh and Dublin graduates were permitted to practice as physicians. There was a curious loophole, apparently in Bristol, indeed, a very unusual one: that the Archbishop of Canterbury could and did frequently in the past issue his licence to practise to physicians. In 1745 I find that a certain John Coopey of Bristol was granted a licence by the

* Doctors of Medicine, 1754 (Harsant) 5: 1794 (Matthews) 14. *Jl.*, 1946, LXIII, p. 92 (Ed.).

Vicar-general of the bishopric: but this is the only one I have found at this time. As a whole the physicians were well educated men of considerable general culture, usually high Tory in politics (inclined to Jacobitism). They tried to make it a rule at the Infirmary that all admissions should be signed by the physician, whether surgical or medical. Eventually after an acrimonious discussion and the threat of wholesale resignations a compromise was effected, or thought to be, by the resolution they passed. " No surgeon shall be allowed to sign any admission ticket when the physician for the week is present but in the physician's absence the surgeon may sign admission tickets for surgical patients." This was apparently rated as a successful compromise but the surgeons contrived to make it a dead letter by keeping the physicians waiting as long as possible.

The physicians dressed for the part in a most dignified way in ruffles and voluminous red cloaks. The red cloak was called a Roquelaure and was almost pathognomonic of the physician. Also a sword was carried. They were required not to live far away so as to be on call at night. It was stated that footpads might prevent their coming from Clifton which had a population of 450 including the Hotwells. The precincts of St. Michael's Hill were praised in a poem in 1712. Mostly they lived in St. James's Barton, round St. Mary Redcliffe and increasingly in Queen Square where Dr. Dover (of powder fame) had one of the first houses built. He had been second in command of a great privateering expedition in 1710 and commanded the captured Spanish galleon. When someone towards the end of the century moved out to Clifton it was doubted as to whether he could properly carry out his duties. Park Street was taken over on lease for building in 1740, but not much was started until 1760.

Physicians' fees were one guinea a visit. We learn this from a complaint as to the stipends of the clergy which averaged £70 per annum: many at £30, most not above £100. It was complained " you do not think a physician overpaid for one guinea a visit but expect to receive a year's services from a clergyman for £5." Two guineas might be charged for a visit to Bedminster or Clifton but never less than one guinea at night. Physicians seem, in some cases, to have been called only to patients *in extremis—nunc dimittis* cases. This is not unusual even to-day. They always felt that musk was required in final prescriptions—so to speak, one could not die without musk (the odour of sanctity). Richard Smith says he often smelt it in the street outside the house of a dying patient. Few could depart without it who could afford it. The bottle cost 12s.—the draft 1s. 6d. and the musk 10s. 6d. How much real knowledge they possessed, how much good they did is very debatable. " A physician is a man who puts into bodies of which he knows little, drugs of which he knows less to cure diseases of which he knows nothing at all " was a jibe which had a certain amount of truth in it. But scan the old records of the B.R.I. and you will be astonished at the number of their cures. Eighty per cent. of the people are stated to have been cured, about 15 per cent. are improved. Results were better than we can fairly and truthfully claim to-day.

Small Pox was a dreaded plague which with each decade of the eighteenth century carried off 10 per cent. of the population and left 20 per cent. disfigured, scarred and blinded. If the infection reached a city, markets stopped, business was at a standstill, the sick were deserted and left to live alone in horror and pain. Every beautiful woman feared lest she should be stricken and left an object for pity rather than for admiration.

[The lecturer next gave an account of the Hotwells.]

The third group of our profession was surgeons. As already pointed out the divorce of the apothecaries from the grocers had already taken place in the seventeenth century and although the College of Physicians strove to keep the apothecaries in their place and a very subordinate one it was, yet by this time the apothecaries had acquired a status of their own and a large degree of independence of the College. But the surgeons were still officially at the beginning of the century " barbers "—barber-surgeons. Some of them practised both branches, e.g. a man called Rosewell to whom was apprenticed William Underhill, who was the first surgeon appointed to the Infirmary. He used to bleed numbers of people on Sundays for a sixpence or a shilling. Another barber in Redcliffe, Henry Haines, used to charge 2d. a shave, 1½d. for a haircut and 6d. a bleeding. Everyone he bled got two quarts of good ale free but you only got a pint with your haircut. One wonders how he managed to make a profit when the brewers' charge was 2d. a gallon. Do I hear a long thirsty sigh rise from the depths of the breasts of some of my audience? Those of us who have attended the efficient bleeding services run by the Blood Donor Organization will remember that while lying down after the removal of our pint our depleted tissues were restored by a cup of strong tea with condensed milk and sugar. Dr. Tovey has learned from two hundred years ago.

But by 1740 the relations of the two wings of the Barbers Company had become very strained. Surgeons were more and more refusing to deal with anything but surgery and in 1739 a number of peruke makers presented a petition to the Barbers' Council complaining of the divers impositions and grievances inflicted on them by the surgical brethren. This was referred to a committee but nothing more was heard of it. Even in those days they knew how efficacious a method this was of avoiding criticism. The company gradually died out as the surgeons refused to belong to it. In 1745 the [London] surgeons petitioned parliament for separation from the barbers and this was granted in June of that year. Soon afterwards [1800] the College of Surgeons came into being as the governing body of the surgeons. The surgeons were apprenticed as students to practising surgeons as private pupils living with the surgeon and assisting him, paying what were in those days good fees. Mr. James Ford, surgeon to the B.R.I. received in thirteen years £708, (below average). The other pupils were Infirmary surgical pupils apprenticed to all the visiting surgeons. These paid each of the five surgeons four guineas per annum and increased greatly in number. In 1758 we find in the records, " Surgeons are allowed to bring apprentices to assist them and allowed to take two pupils and to take money for teaching them ".

The surgeons were undoubtedly men of considerable skill and acumen. Their results were lamentable due to complete ignorance of infection. They operated in old coats, stiff with blood, they stuck the needles in the lapels of their coats, they prided themselves on the filthy condition of their operating coats. They produced, when lucky, " laudable pus " : when unlucky hospital gangrene carried off their patients and that only too often. But when we remember that they had to operate without anaesthetics, to operate in the shortest possible time, to cut for stone by lateral lithotomy on a struggling patient in an ill-lighted room, we can only marvel at their manual dexterity. Their knowledge of anatomy was not very good but they constantly sought to improve it. They dissected the body of every criminal executed: and you could be executed for everything from a theft of about 5s. They also dissected the bodies of those newly buried and paid the people who did the body-snatching. The annals of the Infirmary are full of gruesome albeit amusing stories of the various contretemps which arose between body-snatching students and grave-guarding relatives. Various courses in anatomy were given by the surgeons at which anyone might attend. Page and Ford ran one in 1746 and another was carried on in 1777 apparently not completely successful either financially or otherwise. It was not until well on in the nineteenth century that the Anatomy Act put an end to the scandal of body-snatching, by providing " subjects " for the proper instruction of students. And yet in spite of all the drawbacks under which they laboured they did good work, these surgeons. Those on the Infirmary staff were the cause of the good name of the B.R.I. being carried all over the West country. But the vast majority of them did not have the opportunities or privileges of being members of the B.R.I. Faculty. They bled patients, did minor operations in houses, set fractures, reduced dislocations. Many of them (even Richard Smith, Junior, the famous Infirmary surgeon), to begin with acted also as apothecaries. They were less well educated as a rule, of lower social status and inferior position to the physicians. Yet they seem on the whole to have been most successful and better professional men. It was on the whole a prosperous, busy, rumbustious existence with leisure almost entirely occupied in conviviality. They were a hard-drinking, large-eating race of men.

I began by quoting Trevelyan and I end by returning to him again. He depicts the eighteenth century as a classical age of freedom, development, culture with improved amenities of life, a more tolerant outlook, a higher standard of living, greater power of enjoyment and living longer to enjoy it: and doubtless he is right. In spite of the cheapness then of all the things now so dear, and the more leisured ways of those times, I am profoundly thankful that my own professional life has been lived in the twentieth and not in the eighteenth century.

Books, etc., consulted:

*Bristol Past and Present*, Nicholls, J. F., and Taylor, J., Vol. III, Bristol, 1882.

*The Annals of Bristol in the Eighteenth Century*, Latimer, J., Bristol, 1893.

*A History of the Bristol Royal Infirmary*, Munro Smith, G., Bristol, 1917.

*English Social History*, Trevelyan, G. M., London, 1944.

*English City*, Ralph, E., Brown, H. G. and Redmayne, P., London, 1945.

*Bristol, England*, Brown, H. G., Bristol, 1946.

*The Reputation of the Hotwells (Bristol) as a Health Resort*, L. M. Griffiths. *Jl.*, 1902, XX, 1, 142, 193.

Cf. also *Jl.*, 1899, XVII, 297; 1908, XXVI, 193; 1912, XXX, 327.

